# The Effects of Reinforcement Techniques in Sleeve Gastrectomy and Roux-en-Y Gastric Bypass: Protocol for a Web-Based Survey, Systematic Review, and Meta-Analysis

**DOI:** 10.2196/50677

**Published:** 2023-12-22

**Authors:** Yunhui Xie, Jun Wen, Hongmei Zhu, Yanjun Liu

**Affiliations:** 1 Center of Gastrointestinal and Minimally Invasive Surgery, Department of General Surgery The Third People's Hospital of Chengdu Affiliated Hospital of Southwest Jiaotong University Chengdu China; 2 College of Medicine Southwest Jiaotong University Chengdu China

**Keywords:** protocol, reinforcement technique, sleeve gastrectomy, Roux-en-Y gastric bypass, systematic review, meta-analysis, bariatric surgery, survey, effectiveness, surgical site, blood loss, blood, gastric bypass, gastrectomy

## Abstract

**Background:**

The effects of reinforcement are still controversial in bariatric surgery, and variations may exist in using this technique.

**Objective:**

This protocol describes a study that aims to survey the views of bariatric surgeons on reinforcement techniques and evaluate the effects of applying reinforcement techniques in sleeve gastrectomy (SG) and Roux-en-Y gastric bypass (RYGB).

**Methods:**

This study is composed of 2 parts. Part 1 will investigate the differences of using reinforcement techniques among surgeons worldwide who perform SG or RYGB through a survey. The survey will be conducted by email and social media. Part 2 will evaluate the safety and effectiveness of using omentopexy or staple line reinforcement in SG and RYGB by systematic review and meta-analysis. In this part, literature searches will be performed in English databases, including CENTRAL, EMBASE CINAHL, Web of Science, and PubMed, and Chinese databases, including Wanfang, China National Knowledge Infrastructure, Database of Chinese Technical Periodicals, and Chinese Biological Medicine, from their establishment to November 2023. Randomized controlled trials and case-control studies will be included. The primary outcomes are rates of postoperative bleeding and gastric leakage. The secondary outcomes include anastomotic stenosis, surgical site infection, reoperation, estimated intraoperative blood loss, operative time (minutes), length of hospital stay (days), overall complications, and 30-day mortality. The meta-analysis will be conducted using RevMan 5.4 under the random-effects model, as well as through extensive subgroup and sensitivity analyses. *P* values <0.05 will be considered statistically significant. This study was registered with PROSPERO (Prospective Register of Systematic Reviews) in accordance with the PRISMA-P (Preferred Reporting Items for Systematic Review and Meta-Analysis Protocols).

**Results:**

The results of this study will be published in a peer-reviewed journal. The web-based survey and initial title or abstract review of papers identified by the search strategy will be completed in November 2023. The second round of title or abstract review and downloading of the papers for full-text inclusion will be completed in January 2024. We aim to complete data extraction and meta-analysis by February 2024 and expect to publish the findings by the end of March 2024.

**Conclusions:**

This study aims to investigate the impact of reinforcement techniques on reducing the incidence of postoperative complications in SG and RYGB procedures and provide assistance for standardizing the procedures of SG and RYGB operations for bariatric surgeons.

**Trial Registration:**

PROSPERO CRD42022376438; https://tinyurl.com/2d53uf8n

**International Registered Report Identifier (IRRID):**

PRR1-10.2196/50677

## Introduction

The prevalence of obesity is steadily rising globally, resulting in a decline in the quality of life of those affected and an increase in mortality rates [1-3]. Various methods are available for managing obesity, including behavioral therapy, pharmacological therapy, and bariatric surgery. Metabolic bariatric surgery has long-lasting weight loss effects, and its positive impact on the treatment of obesity-related comorbidities (eg, hypertension, type 2 diabetes, and obstructive sleep apnea) has also been observed [4,5].

Roux-en-Y gastric bypass (RYGB) and sleeve gastrectomy (SG) are the most frequently performed bariatric surgeries [6,7], which typically involve the use of reinforcement techniques to reduce potential postoperative complications, such as gastroesophageal reflux, gastric leakage, and food intolerance. There are variations of reinforcement techniques used by different surgeons and in different countries [8-11], which can be categorized by material (eg, seamguard, peri-strips, fibrin sealant, absorbable polymer membrane, medtronic), suture methods (eg, oversew, burying, and whole layer continuing), the degree of reinforcement (partial or full), and types (staple line reinforcement or omentopexy). These variations may be related to differences in the effectiveness of reinforcement techniques in SG and RYGB, which make it a challenge to decide if a reinforcement technique is needed and which one is most appropriate for the patient (Table 1). For example, in terms of SG operations, 4 systematic reviews [12-15] reported that staple line reinforcement did not have any clear benefit concerning gastric leakage rate, overall morbidity, and mortality rate, while 3 other systematic reviews [16-18] found that staple line reinforcement overperformed in reducing the rate of gastric leakage and bleeding compared to no reinforcement. With regard to RYGB, a meta-analysis of 3 randomized controlled trials found a reduction in the rate of leakage in participants with staple line reinforcement [19], whereas another systematic review demonstrated that the rate of gastric leakage was no different with or without staple line reinforcement [20].

Apart from staple line reinforcement, omentopexy is another type of reinforcement technique, which uses sutures to fix the rest of the stomach to the free edge of the greater omentum [21]. The effect of SG with omentopexy on the rate of postsurgery complications was investigated in 3 systematic reviews [22-24], which reported inconsistent results. Of the 3 reviews, 2 [22,24] suggested that SG with omentopexy outperforms SG without omentopexy in reducing gastrointestinal adverse reactions (eg, nausea, reflux, and vomiting), while in terms of reducing gastric leakage, they showed the opposite effects. Opposing results were also observed in 2 reviews [23,24] in terms of the length of hospital stay.

According to Table 1, we found that previous systematic reviews of reinforcement techniques either focused on a single type of operation (SG or RYGB) or a single type of reinforcement technique (staple line reinforcement or omentopexy), and the design of the included studies was also relatively simple. Thus, an up-to-date and comprehensive meta-analysis and systematic review is needed to fill the gaps and confirm the effect of reinforcement techniques in SG and RYGB. Therefore, a web-based survey to ascertain the variations in the utilization of reinforcement techniques among surgeons worldwide who perform SG or RYGB will be performed in this study, which aims to elucidate the necessity and rationale for conducting the ensuing meta-analysis and systematic review. The goal of these analyses is to investigate the impact of reinforcement techniques on reducing the incidence of postoperative complications in SG and RYGB procedures.

**Table 1:** Summary of previous systematic reviews on reinforcement techniques in sleeve gastrectomy (SG) and Roux-en-Y gastric bypass (RYGB).

Study	Type of surgeries	Type of reinforcement techniques	Design of included studies	Meta-analysis	Subgroup analyses	Sensitivity analyses	Quality assessment	Other analyses	Number of studies included
Giannopoulos et al, 2010 [12]	SG, BPD-DS^a^, RYGB, VBG^b^	SLR^c^	RCTs^d^, non-RCTs	Yes	No	No	No	PB^e^	17
Sajid et al, 2011 [19]	RYGB	SLR	RCTs	Yes	No	No	No	N/A^f^	3
Choi et al, 2012 [20]	SG	SLR	RCTs, Cs^g^	No	No	No	No	N/A	8
Aurora et al, 2012 [13]	SG	SLR	RCTs, Cs	No	No	No	No	No	29
Parikh et al, 2013 [15]	SG	SLR	Retrospective	Yes	No	No	No	LRA^h^	112
Knapps et al, 2013 [14]	SG	SLR	Retrospective, Prospective, RCTs	Yes	Yes	No	No	No	30
Gagner and Buchwald, 2014 [16]	SG	SLR	Retrospective, Prospective, RCTs, No report	No	No	No	No	No	88
Wu et al, 2019 [17]	SG	SLR	RCTs	Yes	Yes	Yes	Yes	PB	11
Zarzycki et al, 2021 [22]	SG	Omentopexy	RCTs, CCSs^i^, Cs	Yes	No	No	Yes	N/A	4
Aiolf et al, 2022 [18]	SG	SLR	RCTs	Yes	No	No	Yes	NMA^j^	17
Chen et al, 2022 [23]	SG	Omentopexy	RCTs	Yes	No	No	No	N/A	5
Wu et al, 2023 [24]	SG	Omentopexy	RCTs	Yes	Yes	Yes	Yes	PB	13
Our protocol	RYGB, SG	SLR, Omentopexy	RCTs, CCSs	Yes	Yes	Yes	Yes	PB, WSBS^k^	50^l^

^a^BPD-DS: biliopancreatic diversion with duodenal switch.

^b^VGB: vertical banded gastroplasty.

^c^SLR: staple line reinforcement.

^d^RTC: randomized controlled trial.

^e^PB: publication bias.

^f^N/A: not applicable.

^g^Cs: cohort study.

^h^LRA: linear regression analysis.

^i^CCS: case-control study.

^j^NMA: network meta-analysis.

^k^WSBS: web-based survey for bariatric surgeons.

^l^Estimated number of eligible studies according to the searching test.

## Methods

**Figure 1:**
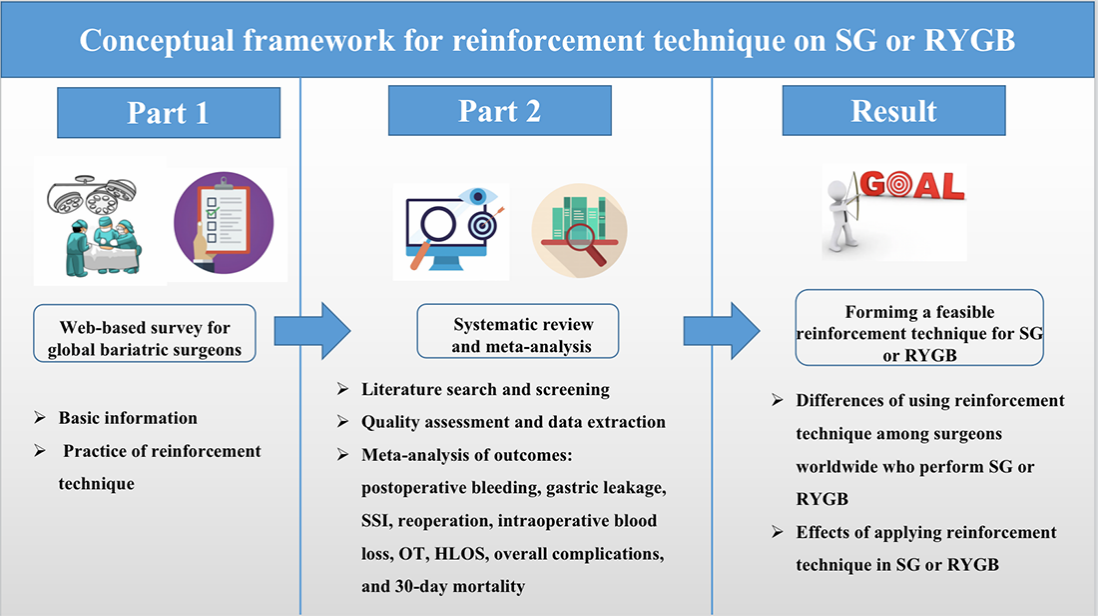
Conceptual framework. HLOS: hospital length of stay; OT: operative time; RYGB: Roux-En-Y gastric bypass; SG: sleeve gastrectomy; SSI: surgical site infection.

This study consists of 2 parts. Part 1 aims to survey global bariatric surgeons via the web and part 2 aims to perform a systematic review and meta-analysis. The specific procedures are illustrated in Figure 1.

**Figure 2:**
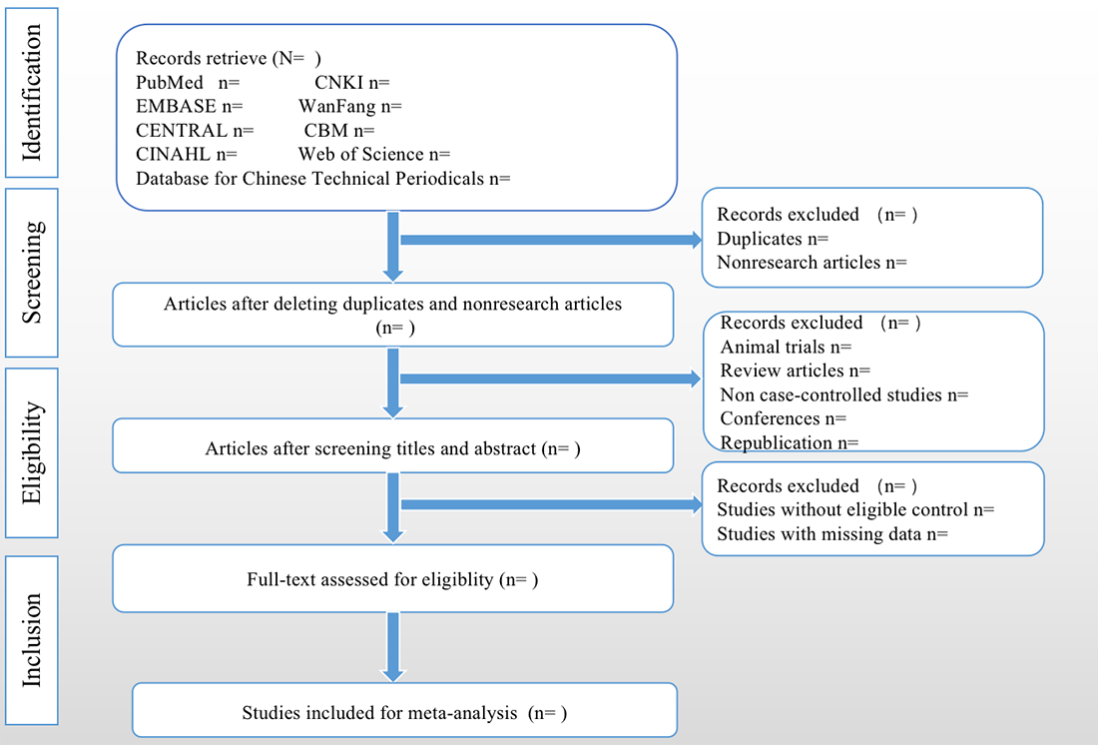
PRISMA flow chart of study.

### Part 1: Web-Based Survey for Global Bariatric Surgeons

A global survey will be conducted using Questionnaire Star to collect basic information and differences in the use of reinforcement techniques among bariatric surgeons from October 30, 2023, to November 30, 2023. The survey includes 6 questions inquiring about basic information, including age, sex, work experience in bariatric surgery, time required for per SG or RYGB surgery (minutes), country, and institution. Additionally, 11 questions pertain to information on reinforcement, including whether to perform SG with staple line reinforcement, the degree of staple line reinforcement in SG, forms of staple line reinforcement in SG, whether to perform SG with omentopexy and their reasons, the degree of omentopexy in SG, whether to perform RYGB with staple line reinforcement and their reasons, the degree of staple line reinforcement in RYGB, and forms of staple line reinforcement in RYGB. The survey will be sent to surgeons through social media (eg, WeChat, Meta) and email. The email addresses of possible surgeons who have published articles in either one of the official journals of The International Federation for the Surgery of Obesity and Metabolic Disorders and the American Society for Metabolic and Bariatric Surgery, namely *Obesity Surgery* and *Surgery for Obesity and Related Diseases*, in the 3-year period from 2020 to 2022 will be captured and used. Only responses from surgeons who perform SG or RYGB will be eligible in later analysis. The survey is shown in Multimedia Appendix 1.

### Part 2: Systematic Review and Meta-Analysis

This systematic review and meta-analysis has been registered on PROSPERO (Prospective Register of Systematic Reviews) with the registration number CRD42022376438. The protocol is reported in accordance with the PRISMA-P (Preferred Reporting Items for Systematic Review and Meta-Analysis Protocols) 2015 [25], which includes the search strategy, eligible criteria, study selection, data extraction and quality assessment, statistical analysis, subgroup analyses, sensitivity analyses, and publication bias.

### Eligibility Criteria

Eligible studies have to meet all of the following inclusion criteria:

1. Population: studies in which all patients underwent SG or RYGB.
2. Intervention: studies in which the intervention was SG or RYGB with omentopexy or staple line reinforcement.
3. Comparison: studies in which the comparison was SG or RYGB without omentopexy or staple line reinforcement.
4. Outcomes: studies that reported at least one of postoperative bleeding and gastric leakage assessment as primary outcomes and secondary outcomes including surgical site infection, reoperation, estimated intraoperative blood loss (milliliters), operative time (minutes), length of hospital stay (days), overall complications, and 30-day mortality. Overall complications are defined as the incidence of peritonitis, abdominal abscess, puncture hernia, intraperitoneal hernia, intestinal adhesion, and gastroesophageal reflux.
5. Study design: randomized controlled trials (RCTs) and case-control studies (CCSs).

Studies will be excluded if they are animal trials or noncontrol studies or do not report sample size and outcome.

### Search Strategy

Searching work will be conducted by 2 researchers (YX and JW) in November 2023. English databases, including CENTRAL, EMBASE CINAHL, Web of Science, and PubMed, and Chinese databases, including Wanfang, China National Knowledge Infrastructure, Database of Chinese Technical Periodicals, and Chinese Biological Medicine, will be searched. The elements like titles, abstracts, and keywords in a combination of medical subject headings (MeSH) terms and free text terms will be searched to ensure an accurate and comprehensive study. The following MeSH and free text terms will be used: gastric bypass, sleeve gastrectomy, omentopexy, and reinforcement. There is no restriction on language and publication date. The detailed search strategy for PubMed is shown in Table 2.

**Table 2:** Search strategy for PubMed.

Search	Query
1	(((gastric bypass[Title/Abstract]) OR (sleeve gastrectomy[Title/Abstract])) OR (SG[Title/Abstract] OR (RYGB[Title/Abstract])
2	(((omentum*[MeSH Terms])) OR (omentopexy[Title/Abstract])) OR (reinforce*[Title/Abstract])
3	(((gastric bypass[Title/Abstract]) OR (sleeve gastrectomy[Title/Abstract])) OR(SG[Title/Abstract] OR (RYGB[Title/Abstract]) AND (((omentum*[MeSH Terms])) OR (omentopexy[Title/Abstract])) OR (reinforce*[Title/Abstract])

### Study Selection and Data Extraction

The literature management software Endnote X9 will be used to retrieve studies and exclude duplicates. The studies’ contents and abstracts will be independently reviewed by 2 authors (YX and JW) according to the precise search strategies after they initially screened the studies that satisfy the inclusion criteria. Data extraction from the included studies, including authors, country, sample size, gender, race, time frame, other comorbidities, type of bariatric operations, reinforcement technique, and reported outcomes, will be collected and managed in Excel (Microsoft Corp; see Multimedia Appendix 2 for a sample table). The full text will then be read by 2 authors (YX and JW) to further determine suitability for inclusion. Any disputes will be settled through discussions or consultation with a third author (HZ).

### Quality Assessment

Quality assessment will be performed based on the study design of the included studies. For RCTs, the Cochrane Risk of Bias tool [26] will be used, which focuses on 6 domains, including randomization, allocation concealment, application of blinding, integrity of the outcome data, selective reporting, and other biases. For CCSs, the quality of the included studies will be assessed by the Newcastle-Ottawa Scale (NOS) [27], which includes the appropriateness of the case and control, representativeness of the case, selection of the control, comparability of the case and control in the design, and statistical analysis of exposure factors. The top score of the NOS is 9, and the higher the score, the higher the quality. Quality assessment will be done by 2 researchers (YX and JW). Any disagreements will be resolved through discussion or consultation with a third researcher (HZ).

### Statistical Analysis

This study will use RevMan 5.4 software (Cochrane) for meta-analysis with the random effects model. We will use odds ratios as the effect measure for dichotomous variables (eg, postoperative bleeding, gastric leakage assessment, and reoperation), and mean differences for continuous variables (eg, estimated intraoperative blood loss, operative time, and length of hospital stay). All effective measures will be accompanied by their corresponding 95% CI. Heterogeneity will be evaluated using the Q test method, which calculates χ^2^ and *I*^2^ values. *P*<0.05 will be considered statistically significant.

### Subgroup Analyses

The following predefined subgroups will be analyzed: study design of the included studies (RCTs or CCSs), age (≤65 years or >65 years) [28], gender (woman or man), type of bariatric surgery (SG or RYGB), materials of staple line reinforcement (eg, seamguard, fibrin sealant, absorbable polymer membrane), suture methods (eg, oversew, burying, whole layer continuing), the degree of reinforcement (partial or full), and the type of reinforcement (staple line reinforcement or omentopexy).

### Sensitivity Analyses

Sensitivity analysis will be performed to assess the robustness of the findings using the leave one out approach [17], and studies involved in this meta-analysis will be removed in turn. Sensitivity analyses on the quality and sample size of the included studies will be conducted as well, and the meta-analyses will be repeated after excluding studies whose sample sizes are <5 or those with 3 domains rated as high risk for RCTs or with a NOS score <5 for CCSs.

### Publication Bias

Publication bias in this study will be assessed using a funnel plot. A Begg test [29] and Egger test [30] will be performed using the Stata 15 software (StataCorp) to evaluate the statistical significance of the publication bias.

### Ethical Considerations

The web-based survey has obtained ethics approval (2023-S-25). As for systematic reviews and meta-analyses, ethics approval is not required because the data used are derived from published papers. Informed consent was not applicable.

## Results

The results of this study will be published in a peer-reviewed journal. The web-based survey and initial title or abstract review identified by the search strategy will be completed in November 2023. The second round of title or abstract review and downloading of the papers for full-text inclusion will be completed in January 2024. We aim to complete the data extraction and meta-analysis by February 2024 and expect to publish the findings by the end of March 2024.

## Discussion

### Principal Findings and Implications

Obesity is a disease caused by excessive accumulation of body fat due to the imbalance between energy intake and consumption [31], which is defined as a body mass index above 30 kg/m^2^ [32]. According to the World Health Organization, more than 1.9 billion people worldwide were overweight in 2016, and more than 650 million had obesity [33]. Bariatric surgery associated with a reduction of obesity-related comorbidities has been gradually recognized globally [34,35]. However, the application of reinforcement techniques in SG or RYGB bariatric surgery remains a challenge for surgeons. In this study, we aim to investigate the opinions of bariatric surgeons on reinforcement techniques using web-based surveys and to evaluate the effectiveness of reinforcement techniques in reducing postoperative complication rates by meta-analyses and systematic review. To our knowledge, few studies have investigated the impact of different reinforcement techniques (staple line reinforcement or omentopexy) on 2 types of bariatric surgery (SG or RYGB) and included 2 study designs (RCT or CCS); these represent the strengths of our study. We will also perform subgroup analysis according to study design, age, race, type of bariatric surgery, materials of staple line reinforcement, suture methods, the degree of reinforcement, and types of reinforcement techniques. Our research will fill previous gaps and help standardize SG and RYGB procedures for bariatric surgeons and other researchers.

### Limitations

Our study has several limitations. First, given the broad inclusion criteria, there may be high heterogeneity among the included studies. We will minimize the potential heterogeneity by performing subgroup and sensitivity analyses. Second, due to language limitations, we will only search English and Chinese databases. We will search for references included in studies to reduce the issue of incomplete inclusion of literature due to language limitations. Third, we are aware that the email addresses obtained from the studies may include authors who are not bariatric surgeons. Therefore, we have indicated on the questionnaire that participants must be bariatric surgeons for subsequent questions to be answered.

### Conclusions

The web-based survey is designed to reveal variations in reinforcement techniques used by bariatric surgeons in SG and RYGB procedures, while the meta-analyses and systematic review are expected to shed light on the effectiveness of these techniques in reducing the incidence of postoperative complications.

## Appendix

Multimedia Appendix 1 Supplementary file.

Multimedia Appendix 2 Sample table.

## Abbreviations

CCS: case-control study
NOS: Newcastle-Ottawa Scale
PRISMA-P: Preferred Reporting Items for Systematic Review and Meta-Analysis Protocols
PROSPERO: Prospective Register of Systematic Reviews
RTC: randomized controlled trial
RYGB: Roux-en-Y gastric bypass
SG: sleeve gastrectomy

## Data Availability

The data sets generated and analyzed throughout the study will be provided as supplementary files in a peer-reviewed journal. Additionally, detailed descriptions and comprehensive analyses of the data will be included to enhance the understanding of the research findings for the bariatric discipline.
