# Supplementary material for: The Effects of Reinforcement Techniques in Sleeve Gastrectomy and Roux-en-Y Gastric Bypass: Protocol for a Web-Based Survey, Systematic Review, and Meta-Analysis
Source: JMIR Res Protoc. 2023 Dec 22;12:e50677. doi: 10.2196/50677 (PMC10770791; doi:10.2196/50677)
Supplement: Multimedia Appendix 1 [file resprot_v12i1e50677_app1.docx]

# Supplementary file

## 1 Questionnaire about staple line reinforcement of SG and RYGB

Dear Surgeon,

We are from Centre of Gastrointestinal and Minimally Invasive Surgery, Department of General Surgery, Third People’s Hospital of Chengdu, who are interested in the variation in practices concerning staple line reinforcement of sleeve gastrectomy (SG) and Roux-en-Y gastric bypass (RYGB). In order to better understand these variations in practices, we would like to invite you to participant in this survey. Your valuable comments and suggestions are critical to us. Thanks for your supporting. All personal information obtained from this survey will be kept strictly confidential and data will only be used for research analysis and reporting only.

Please confirm that you are a Bariatric Surgeon who performs SG or RYGB.

1.Gender □Male □Female

2.Age

3.How many years have you performed bariatric surgery to date?

4.Approximately how many SG or RYGB procedures have you performed to date? (Please fill in the quantities of LSG and LRYGB respectively and separate them with symbols, e.g., ‘60/70’, ‘60, 70’, ‘60; 70’, etc.)

5.Approximately how many minutes do you need for performing a SG or RYGB procedure? (Please fill in the times of performing LSG and LRYGB respectively and separate them with symbols, e.g., ‘60/70’, ‘60, 70’, ‘60; 70’, etc.)

6.Which country do you work in

7.The hospital you work for belongs to (Multiple choices)

□Teaching and research hospital

□Private hospital

□Military hospital

□Clinic

□State hospital

□Municipal hospital

□Others___________(Please specify)

1. Do you routinely perform LSG with staple line reinforcement?

□Yes □No □Not applicable(Yes, skip to question 2; No, skip to question 4; No applicable, skip to question 8)

2.The degree of staple line reinforcement is?

□Partial reinforcement □Full reinforcement

3.The form(s) of staple line reinforcement you use (Whatever choose, skip to question 5)

□Seamguard □Oversew using a running suture □Burying (invaginating) using a running suture □Whole layer continuing using a running suture □Lambert suture □ Peri-Strips □Fibrin sealant □ Medtronic □Others___________(Please specify)

4.The reason you choose not to use staple line reinforcement is based on (Whatever choose, skip to question 5)

□ Prolonged operation time

□ High tension at suture

□ Narrowed stomach cavity

□ Others___________(Please specify)

5.Do you routinely perform SG with omentopexy?

□Yes □No (Yes, skip to question 6; No, skip to question 7)

6.The degree of omentopexy is?

□Partial omentopexy □Full omentopexy (Whatever choose, skip to question 8)

7.The reason you choose not to perform omentopexy is based on

□ Prolonged operation time

□ High tension at suture

□ Unable to restore to normal anatomical state

□ Others___________(Please specify)

8.Do you routinely perform LRYGB with staple line reinforcement?

□Yes □No (Yes, skip to question 9; No, skip to question11)

9.The degree of staple line reinforcement is?

□Partial reinforcement □Full reinforcement

10.The form(s) of staple line reinforcement you use (Whatever choose, end of questionnaire)

□Seamguard □Oversew using a running suture □Burying (invaginating) using a running suture □Whole layer continuing using a running suture □Lambert suture □ Peri-Strips □Fibrin sealant □ Medtronic □Others___________(Please specify)

11.The reason you choose not to use staple line reinforcement is based on (Whatever choose, end of questionnaire)

□ Prolonged operation time

□ High tension at suture

□ Narrowed stomach cavity

□ Others___________(Please specify)
