# Supplementary material for: The Effects of Reinforcement Techniques in Sleeve Gastrectomy and Roux-en-Y Gastric Bypass: Protocol for a Web-Based Survey, Systematic Review, and Meta-Analysis
Source: JMIR Res Protoc. 2023 Dec 22;12:e50677. doi: 10.2196/50677 (PMC10770791; doi:10.2196/50677)
Supplement: Multimedia Appendix 2 [file resprot_v12i1e50677_app2.docx]

Sample Table. Characteristics of included studies.

| Study | Study  design | Country | Sample size  (I/C) | Gender  (I/C:  (female/male) | Race | Time frame | Other comorbidities | Type of bariatic operation | Reinforcement technique | | | | Reported outcomes | | |
| --- | --- | --- | --- | --- | --- | --- | --- | --- | --- | --- | --- | --- | --- | --- | --- |
|  |  |  |  |  |  |  |  |  | Materials | Suture methods | Degree | Type | No. of postoperative bleeding  (I/C) | No. of gastric leakage  (I/C) | Other secondary outcomes(I/C) |
| Study 1 |  |  |  |  |  |  |  |  |  |  |  |  |  |  |  |
| Study 2 |  |  |  |  |  |  |  |  |  |  |  |  |  |  |  |
| Study 3 |  |  |  |  |  |  |  |  |  |  |  |  |  |  |  |
| …… |  |  |  |  |  |  |  |  |  |  |  |  |  |  |  |

For RCTs: I: intervention; C: control
